# Effect of Muscle Extract and Graphene Oxide on Muscle Structure of Chicken Embryos

**DOI:** 10.3390/ani11123467

**Published:** 2021-12-06

**Authors:** Jaśmina Bałaban, Marlena Zielińska, Mateusz Wierzbicki, Teresa Ostaszewska, Magdalena Fajkowska, Małgorzata Rzepakowska, Karolina Daniluk, Malwina Sosnowska, André Chwalibog, Ewa Sawosz

**Affiliations:** 1 Department of Nanobiotechnology, Institute of Biology, Warsaw University of Life Sciences, 02-786 Warsaw, Poland; jasmina_balaban@sggw.edu.pl (J.B.); marlena_zielinska@sggw.edu.pl (M.Z.); mateusz_wierzbicki@sggw.edu.pl (M.W.); karolina_daniluk@sggw.edu.pl (K.D.); malwina_sosnowska@sggw.edu.pl (M.S.); ewa_sawosz_chwallibog@sggw.edu.pl (E.S.); 2 Department of Ichthyology and Biotechnology in Aquaculture, Institute of Animal Science, Warsaw University of Life Sciences, 02-786 Warsaw, Poland; teresa_ostaszewska@sggw.edu.pl (T.O.); magdalena_fajkowska@sggw.edu.pl (M.F.); malgorzata_rzepakowska@sggw.edu.pl (M.R.); 3 Department of Veterinary and Animal Sciences, University of Copenhagen, 1870 Frederiksberg, Denmark

**Keywords:** chicken embryo, development, in ovo, graphene oxide, histology, muscle, toxicity

## Abstract

**Simple Summary:**

Genetic selection of broilers increased muscle growth; however, very fast growth can lead to pathological conditions caused by the deficiency of nutrients. The number of muscle cells is mainly formed during the embryonic period, and consequently, in ovo supplementation of proteins to embryos may impact future muscle structure. We hypothesized that proteins from chicken embryo muscle extract (CEME) caused by the unique, natural composition and biocompatibility can supply additional proteins. However, supplemented proteins are actively metabolized, which may reduce their utilization for improved muscle synthesis. Nevertheless, CEME can be transported and protected by graphene oxide (GO). The objective of the present work was to investigate the effects of in ovo-injected CEME and the complex of GO-CEME on embryonic cell cultures and the growth of chicken embryo hind limb muscle. Toxicity and cell proliferation were measured in vitro with cell cultures and mortality, morphology, histology, and blood biochemistry in vivo with embryos. CEME increased the number of cells and nuclei in muscle, but the complex GO-CEME did not further improve the muscle structure. The results indicate a vital role of CEME as in ovo enhancer of muscle development in broilers.

**Abstract:**

The effects of CEME and it complex with GO injected in ovo on the growth and development of chicken embryo hindlimb muscle were investigated. First, the preliminary in vitro study on primary muscle precursor cell culture obtained from a nine-day-old chicken embryo was performed to assess toxicity (MTT assay) of CEME, GO (100 ppm) and it complex with different concentrations (1, 2, 5, and 10 wt.%). The effect on cell proliferation was investigated by BrdU assay. CEME at concentrations 1–5% increased cell proliferation, but not the complex with GO. In vitro cytotoxicity was highest in 10% and GO groups. Next, the main experiment with chicken embryos was performed with CEME, GO and it complex injected in ovo on day one of embryogenesis. On day 20 of embryogenesis survival, morphological development, histological structure of the muscle, and biochemical parameters of blood serum of the embryos were measured. No negative effect on mortality, body weight, or biochemistry of blood after use of CEME or GO-CEME complexes was observed. Interestingly, the slight toxicity of GO, observed in in vitro studies, was not observed in vivo. The use of CEME at the levels of 2% and 5% improved the structure of the lower limb muscle by increasing the number of cells, and the administration of 2% CEME increased the number of nuclei visible in the stained cross-section of the muscle. The complex GO-CEME did not further improve the muscle structure. The results indicate that CEME can be applied as an in ovo enhancer of muscle development in broilers.

## 1. Introduction

Genetic selection has increased poultry meat production, but it has been followed by substantial deterioration of the muscle structure. Comparing the muscles of broiler chickens to laying hens that were not selected for the increase of the pectoral muscle, a much larger number of muscle fibers and an increase in their diameter are observed [1]. Analysis of the breast muscle genes showed an increase in the expression of genes responsible for the development of free-type muscle fibers, the proliferation of satellite cells, as well as muscle hypertrophy in broiler chickens compared to laying hens [2]. Consequently, a very fast growth rate of broiler chicken can lead to disturbance of the normal structure of the muscle tissue, especially the pectoral muscles, which account for 31% of body weight [3]. The more and more frequent pathological conditions of the muscles, and above all myopathies of the pectoral muscle, regardless of the etiology, to a large extent may result from the deficiency of certain structural and functional proteins. Disruption of the correct ratio of proteins involved in the proliferation, differentiation, maturation of muscle fibers, and the final formation of its structure may predispose to its weakening or even dystrophy. Muscle degeneration is usually associated with an accumulation of interstitial connective tissue or fibrosis [4], resulting from impaired synthesis of amino acids and proteins. Moreover, the pool of exogenous amino acids is limited due to the development of the chicken embryo outside the mother’s organism. On the other hand, the proliferation of muscle cells decreases before hatching so that the number of muscle cells is largely formed during the embryonic period. Therefore, only in ovo supplementation can have a decisive impact on the formation of the future correct muscle structure.

The studies showed that the in ovo administration of the amino acid composition to the embryo [5] increased the growth of chickens. Taurine, supplemented in ovo to chicken embryos, improved the structure of breast muscle by increasing the expression of PCNA protein [6]. L-Arginina conjugated with nanoparticles of Ag activates myogenin and MyoD expression, which is pointed to improve muscle growth [7]. Due to the fact that embryonic development takes more than 1/3 of the life of broiler chickens and is a key period for muscle development, the method of in ovo feeding is very promising [8]. However, it entails a number of difficulties, and the key ones include the injection sterility regime and the too rapid degradation of nutritional–functional ingredients delivered. Moreover, the rapid catabolism of supplemented protein may have negative effects resulting from the accumulation of harmful products of protein breakdown. Therefore, the optimization of in ovo supplementation should take into account not only the appropriate composition and the dose of administered compounds but also their metabolism over time.

In previous in vitro studies, we used chicken embryo muscle extract (CEME) extract from an 18-day-old chicken embryo to cultivate muscle tissue from muscle precursor cells taken from a limb bud on day eight of embryogenesis [9]. The results of the research showed that this extract stimulated not only the formation of the correct structure of pseudo-muscle tissue but also its physiological maturation, observed as contractions characteristic of muscle tissue. It should be emphasized that these contractions occurred without any external electrical stimulation but only after administration of the CEME extract—a cocktail of a wide variety of proteins. This interesting observation inspired us to expand our research and determine whether this cocktail could be used not only for tissue culture in vitro but also as a stimulator of muscle development when administered to the chicken embryo in ovo. It has to be underlined that CEME and the complex of CEME with graphene oxide (GO) (GO-CEME) have not been used previously.

Proteins administered to the body from the outside are actively metabolized, which may reduce their utilization and promote the formation of protein degradation products. To counteract such adverse effects, CEME extract was conjugated with GO and administered in the form of a GO-CEME complex. The form of the complex would protect the extract against rapid decomposition and ensure a slow release of extract proteins. Moreover, GO is a carbon structure—a graphite derivative—which, when administered at an appropriate level, is non-toxic and biocompatible [10,11,12,13]. Therefore, research on GO is dedicated to using it to create scaffolds in tissue engineering [14,15] or to transport drugs or functional compounds in medicine [16]. The huge surface area of graphene flakes and the available chemical groups favor the formation of the protein crown as a result of self-organization on the GO surface, especially in the presence of a large number of proteins [17]. The protein crown is an active structure, and its composition changes depending on the physicochemical conditions of the biological environment. This very dynamic process allows the use of GO as a carrier in drug delivery systems. We supposed that CEME could be transported on GO flakes, increasing the activity of the protein cocktail. Taking into account the CEME activity observed in previous in vitro studies [9] and the transport properties of GO, we decided to create a “nutrient delivery” system similar to the “drug delivery” systems used in medicine. The present model studies were aimed at checking the possibility of modifying the development and shaping the structure of striated muscle by “feeding” the embryo. The planned feeding system was to meet two conditions: 1. be spread over time and minimize uncontrolled breakdown of nutrients, 2. be the most comprehensive source of nutrients. Consequently, the main objective was to determine whether the extract from embryo muscle conjugated with GO is not toxic and can stimulate the formation of a better muscle structure of embryos, and in the future, muscle development of broilers. The findings can also be utilized in the area of muscle regeneration in animals and humans.

## 2. Materials and Methods

### 2.1. Graphene Oxide

Single layer of graphene oxide, as water dispersion 2 wt.%, was purchased from US Research Nanomaterials, Inc. (Houston, TX, USA). GO flakes had thickness of 0.43–1.23 nm and diameter of 1.5–5.5 μm (manufacturer’s data).

The morphology of GO was characterized using a TEM-JEM-1220 (JEOL, Tokyo, Japan) at 80 kV and a TEM CCD Morada 11-megapixel camera (Olympus Soft Imaging Solutions, Munster, Germany). The zeta potential of GO solution was measured by light scattering using a ZetaSizer Nano ZS model ZEN3500 (Malvern Instruments, Malvern, UK). All measurements were performed in four repetitions. For the experiment, GO was diluted in ultrapure Milli-Q water at the final concentration of 100 ppm and sonicated in an ultrasonic bath (Bandelin Electronic, Berlin, Germany).

### 2.2. Preparation and Analysis of Chicken Embryo Muscle Extract

Fertilized chicken (Ross 308) eggs were purchased from a commercial hatchery, sterilized in KMnO_4_ solution, irradiated with UV, and incubated at 37 °C and ~60% relative humidity. The muscle tissue was obtained from a 20-day-old chicken embryo. After decapitation of the embryo, the explant from the hindlimb was dissected. The skin and bones were removed from the sample. A sample of muscle tissue was suspended in ultrapure water and homogenized with frozen metal balls in a TissueLyser ball mill (Qiagen, New York, NY, USA). The homogenate was centrifuged at 1400× *g* for 45 min at 4 °C. The supernatant was stored at −80 °C.

The protein composition of the extract was analyzed by mass spectrometry (MS) using an Orbitrap Elite (Thermo Fisher Scientific, Massachusetts, MA, USA) connected to a Water Acquity HPLC, according to the methodology described in the previous article [9]. Obtained data were deposited in the PRIDE repository under PXD015146. The top 100 protein identified in the CEME was grouped on the proteins associated with cell proliferation, metabolism, extracellular matrix organization, nervous system, etc. The protein–protein interaction network analysis was performed in inBio Discover™.

The GO-CEME complex was prepared by mixing GO solution in concentration of 100 ppm with adequate addition of CEME solution and sonicated in an ultrasonic bath (Bandelin Electronic, Berlin, Germany) for self-organization.

### 2.3. In Vitro Study: Cell Culture

Muscle precursor cells were obtained from 9-day-old chicken embryo by dissection of the hindlimb. The sample was treated with trypsin for 24 h at 4 °C and disintegrated by gentle pipetting. After that, cells were seeded onto a culture plate. Cells were maintained in Dulbecco’s Modified Eagle’s medium (DMEM) (Life Technologies, Texas, TX, USA) supplemented with 10% fetal bovine serum (Life Technologies, Texas, TX, USA) and 1% penicillin/streptomycin (Life Technologies, Texas, TX, USA) at 37 °C in a humidified atmosphere of 5% CO_2_/95% air in a Memmert ICO150med Incubator (Memmert, Schwabach, Germany). Cells were divided into experimental groups: (1) Control (not treated), (2) CEME0.1%, (3) CEME0.2%, (4) CEME0.5%, (5) CEME1%, (6) CEME2%, (7) CEME5%, (8) CEME10%, (9) GO (100ppm), (10) GO-CEME0.1%, (11) GO-CEME0.2%, (12) GO- CEME0.5%, (13) GO-CEME1%, (14) GO-CEME2%, (15) GO-CEME5%, and (16) GO-CEME10%. In all groups with GO was added 10% of culture media total volume with constant concentration (100 μg/L) and adequate addition of CEME (0.1%, 0.2%, 0.5%, 1%, 2%, 5%, 10% of culture media total volume).

### 2.4. In Vitro Study: Proliferation of Cells

Proliferation status of primary cell culture was tested with a Cell Proliferation ELISA, BrdU (colorimetric) kit (Roche Applied Science, Indianapolis, IN, USA). After 24 h of maintaining cells with experimental complexes (as described in section *Cell Culture*), BrdU reagent (10 μL) was added to the culture media, cells were stained for 4 h. All further steps proceeded according to the manufacturer’s instructions. The results were analyzed by reading absorbance at 370 nm with a reference wavelength of 492 nm using Tecan Infinite 200 plate reader (Tecan, North Carolina, ND, USA.).

### 2.5. In Vitro Study: Viability of Cells

Viability was tested with a Vybrant Cell Proliferation Assay Kit (MTT) (Thermo Fisher Scientific, Massachusetts, MA, USA) after 24 h of treatment with experimental complexes according to the scheme described in section *Cell Culture*. Cells were cultured in 96-well plates. MTT reagent (10 μL) was added to each well and incubated for 3 h at 37 °C. Next, cells were disintegrated with lysis buffer (containing Isopropanol, Triton X, and HCl). Culture plates were centrifuged, and the supernatant was transferred to new 96-well plates. The absorbance of samples (n = 6) was measured using Tecan Infinite 200 plate reader (Tecan, North Carolina, ND, USA.) at 570 nm according to the manufacturer’s instructions.

### 2.6. Hemotoxicity

The hemolysis assay was performed on the chicken blood. The whole blood sample was centrifuged for 5 min at 5000 rpm and plasma and lymphocytes were removed. Fraction of red blood cells were washed with PBS and centrifuged 3 times at 150× *g* for 5 min. The final pellet of red blood cells was suspended in PBS. Experimental complexes (0.1 mL) were added to the blood samples (1 mL) and incubated for 1 h at 37 °C. Negative control (0% hemolysis) was treated with PBS and positive control (100% hemolysis) was treated with 20% solution of Triton-X. After the incubation, all samples were centrifuged at 900× *g* for 10 min, 200 μL of supernatant were transferred to the new 96-well plate, and absorbance at 405/540 nm (Infinite M200, Tecan, Durham, NC, USA) was measured.

### 2.7. Animal Model

Eggs were randomly divided into 12 groups: (1) Control (not treated), (2) CEME1%, (3) CEME2%, (4) CEME5%, (5) CEME10%, (8) GO, (9) GO-CEME1%, (10) GO-CEME2%, (11) GO-CEME5%, and (12) GO-CEME10%. The experimental solution complexes were injected in ovo (200 μL) on the first day of incubation. The injection was performed into the albumen with a sterile tuberculin syringe. The injection site was secured with sterile tape, and the eggs were placed in an incubator. The embryos were observed on the 20th day of incubation. Mass of eggs and the embryos were measured and the development of the embryos was assessed. Embryos were sacrificed by decapitation, and the blood samples, liver, and heart were collected and the organs were weighed. Blood samples were analyzed for albumins, alanine transaminase (ALT), alkaline phosphatase (ALP), aspartate transaminase (AST), total protein, creatinine, urea, globulins, lactate dehydrogenase (LDH), glucose, and triglyceride by a commercial diagnostic veterinary laboratory (VetLab, Warsaw, Poland) using standard diagnostic kits and protocol.

### 2.8. Immunochistochemistry

For histological and immunohistochemical analysis, tissues were fixed in 4% buffered formalin. Formalin-fixed muscle samples were paraffin-embedded into blocks by the standard paraffin technique and cut using Rotary Microtome HM 355 S (Microm, Walldorf, Germany) with section transfer system STS. Tissue sections were stained using Periodic Acid–Schiff (PAS) Staining System (Sigma-Aldrich, St. Louis, MO, USA) according to the manufacturer’s instructions. All the sections were observed and recorded using a Nikon Eclipse Ni light microscope with Nikon DS-Fi3 camera (Nikon Corporation, Tokyo, Japan). The number of cells per microscopic field of view (n = 5; area: 0.09 mm^2^) was calculated manually using ImageJ-Cell Counter. Fields of view were chosen randomly.

For PCNA detection, monoclonal, mouse, PCNA antibody (No. 13–3900, Life Technologies Rockford, IL, USA.) in a 1:50 dilution was applied on tissue sections. Incubation with anti-PCNA antibody was performed for 60 min at RT. A citrate buffer was used for Heat-Induced Epitope Retrieval (HIER). A Peroxidase Detection System (Dako North America, CA, USA) was applied for visualization in accordance with manufacturer recommendations. Samples were counterstained with Harris Hematoxylin (Mar-four, Konstantynów Łódzki, Poland). Images of stained sections were analyzed by ImageJ-Cell Counter, PCNA-positive and PCNA-negative nuclei were counted manually on randomly selected microscopic fields of view (n = 5; area: 0.09 mm^2^), and the percentage of PCNA-positive nuclei of the total nuclei number (PCNA-positive and PCNA-negative) was presented as Proliferation Index.

### 2.9. Superoxide Dismutase (SOD) Activity

Superoxide dismutase (SOD) Assay Kit (Sigma-Aldrich, St. Louis, MO, USA) was used to determine SOD activity in homogenized liver samples. The samples (10 mg, n = 3 per group) were suspended in ultrapure water/PBS and homogenized with frozen metal balls in a TissueLyser ball mill (Qiagen, New York, NY, USA). The homogenate was centrifuged at 1000× *g* for 20 min. The total protein content in supernatant after homogenization was determined using a BCA Protein Assay Kit according to the manufacturer’s protocol (Sigma-Aldrich, St. Louis, MO, USA). SOD Assay Kit (Sigma-Aldrich, St. Louis, MO, USA) was performed according to the manufacturer’s protocol.

### 2.10. Statistical Analysis

Data were analyzed using monofactorial analysis of variance, ANOVA. Differences between groups were tested with multiple-range Tukey’s test using software StatGraphics Centurion version XVI (StatPoint Technologies, Georgia, GA, USA). The significant differences between groups were marked with different letters (a, b, and c). Differences with *p* < 0.05 were considered significant.

## 3. Results

### 3.1. GO and CEME Characterization

**Figure 1:**
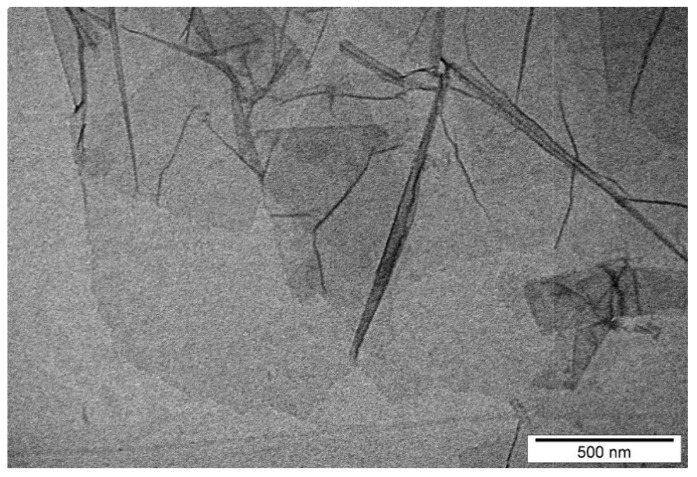
Transmission electron microscopic images of graphene oxide flakes.

The zeta potential of GO solution in a concentration of 100 ppm was 38.8 mV. Transmission Electron Microscopy (TEM) imaging was used to visualization the morphology of the flakes of GO (Figure 1).

**Figure 2:**
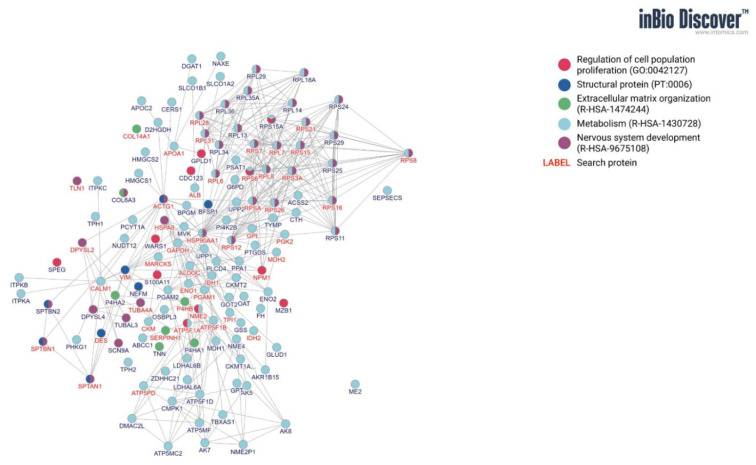
The protein-protein network of top 100 proteins identified in CEME using Mass Spectrometry.

The detailed protein profile of the aqueous CEME extract was presented in the previous publication of the authors [9]. This article additionally presents the protein–protein interaction network of the top 100 proteins of the extract visualized by inBio Discover™, taking into account categories, regulation of proliferation (positive vs. negative), and functions (ECM organization, nervous system development, and metabolism) (Figure 2). The largest group (67) are proteins involved and co-involved in proliferation, the second (35) are proteins related to metabolism, 23 proteins are related to the functioning of the nervous system, 3 proteins participated in the organization of ECM, and 5 are structural proteins.

The GO and CEME complexes formed stable, colloidal structures. No visible aggregates or sediment were observed.

### 3.2. In Vitro Study: Proliferation of Cells

**Figure 3:**
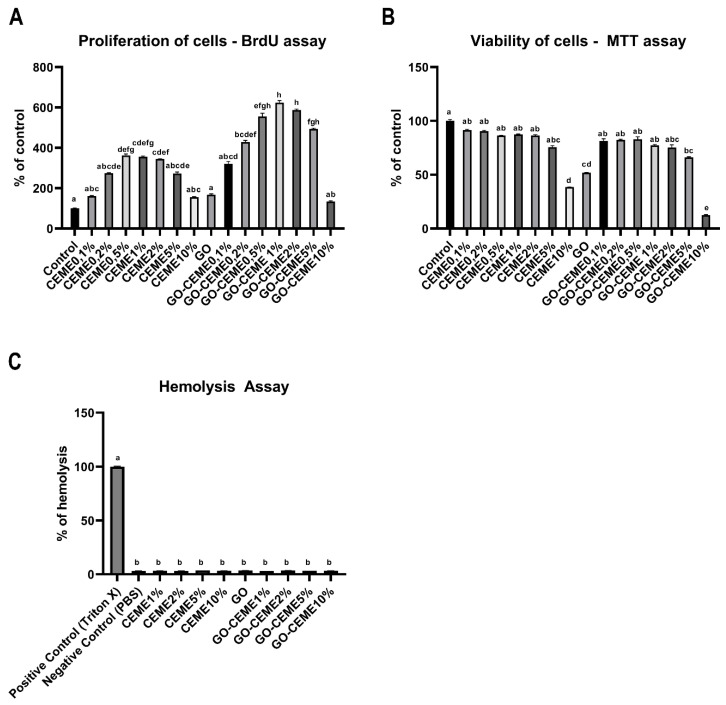
(**A**) Proliferation of muscle precursor cells from 9-day-old chicken embryo measured by BrdU assay; (**B**) Viability of muscle precursor cells from 9-day-old chicken embryo measured by MTT assay; (**C**) Results of hemolysis assay performed on chicken red blood cells; positive control (Triton X)–100% of hemolysis of red blood cells. Presented as mean value with standard deviation, different letters denote significant difference, *p* < 0.05.

Cell proliferation was analyzed with the BrdU assay based on the quantitative measurement of DNA synthesis by detecting the thymidine nucleoside analog incorporated into the DNA of dividing cells during the S phase. The obtained results showed that the CEME extract, administered at 0.5%, 1%, and 2%, increased the proliferation of muscle cells. GO applied to the medium did not affect the proliferation of cells. However, administration of CEME in a complex with GO (GO-CEME1%; GO-CEME2%; GO-CEME5%) resulted in a significant increase in cell proliferation compared to the administration of the extract alone (Figure 3A).

#### 3.2.1. In Vitro Study: Viability of Cells

The in vitro cytotoxicity study of GO and CEME was performed by the MTT method by determining the metabolic activity of cells (mitochondrial dehydrogenase activity). The results showed the toxicity of CEME10%, GO and GO-CEME10% and to a small extent, GO-CEME5% (Figure 3B). Moreover, the GO-CEME10% complex had a more negative effect compared to CEME10% as well as GO.

#### 3.2.2. Hemolysis Assay

The basic test of the biocompatibility of nanomaterials is the hemocompatibility test, which determines the potential hemolysis of blood cells after contact with the tested material. No deleterious effects of GO, CEME, and also GO, complexed with CEME up to 10% (Figure 3C), on cell membrane stability and hemolysis after 15, 30, and 60 min were observed.

### 3.3. In Ovo Study: Chicken Embryo Growth and Development

GO, CEME (in concentrations of 1%, 2%, 5%, and 10%) and GO with CEME complexes (GO-CEME1%, GO-CEME2% GO-CEME5%, GO-CEME10%) were administered to the chicken embryo at the beginning embryogenesis. The mean egg mass was not significantly different between groups (*p* > 0.05). At the end of embryogenesis (day 20), the growth and development of the embryos were assessed. The administered GO, CEME, and GO-CEME complexes did not reduce the survival rate of the embryos. Moreover, the bodyweight of the embryos and the relative weight of the heart, liver did not change in relation to the control group (Table 1). During the necropsy, no visible malformations, genetic or other pathological changes of the embryos were found.

On day 20 of embryogenesis, blood was collected from the embryos, and the basic blood biochemical parameters were determined (Table 2). The analysis of selected blood biochemical parameters of embryos showed no effect of GO, CEME, and GO-CEME complexes on the activity of the liver enzymes ALT, AP, AST, and LDH as well as on the concentration of albumin, total protein, globulin, TG, and creatinine. There was a slight trend of urea increase in the groups receiving GO and CEME and a decrease in urea after administration of the GO-CEME complex. However, only statistically significant differences were found between GO, CEME5%, and CEME10% vs. GO-CEME5% (Table 2). A reduction in blood glucose concentration was also observed with the administration of GO, CEME10%, and GO-CEME10%. However, the observed differences were not significant, and all results were in the reference ranges.

### 3.4. In Ovo Study: Morphological and Immunohistochemical Evaluation of Muscle Structure

**Figure 4:**
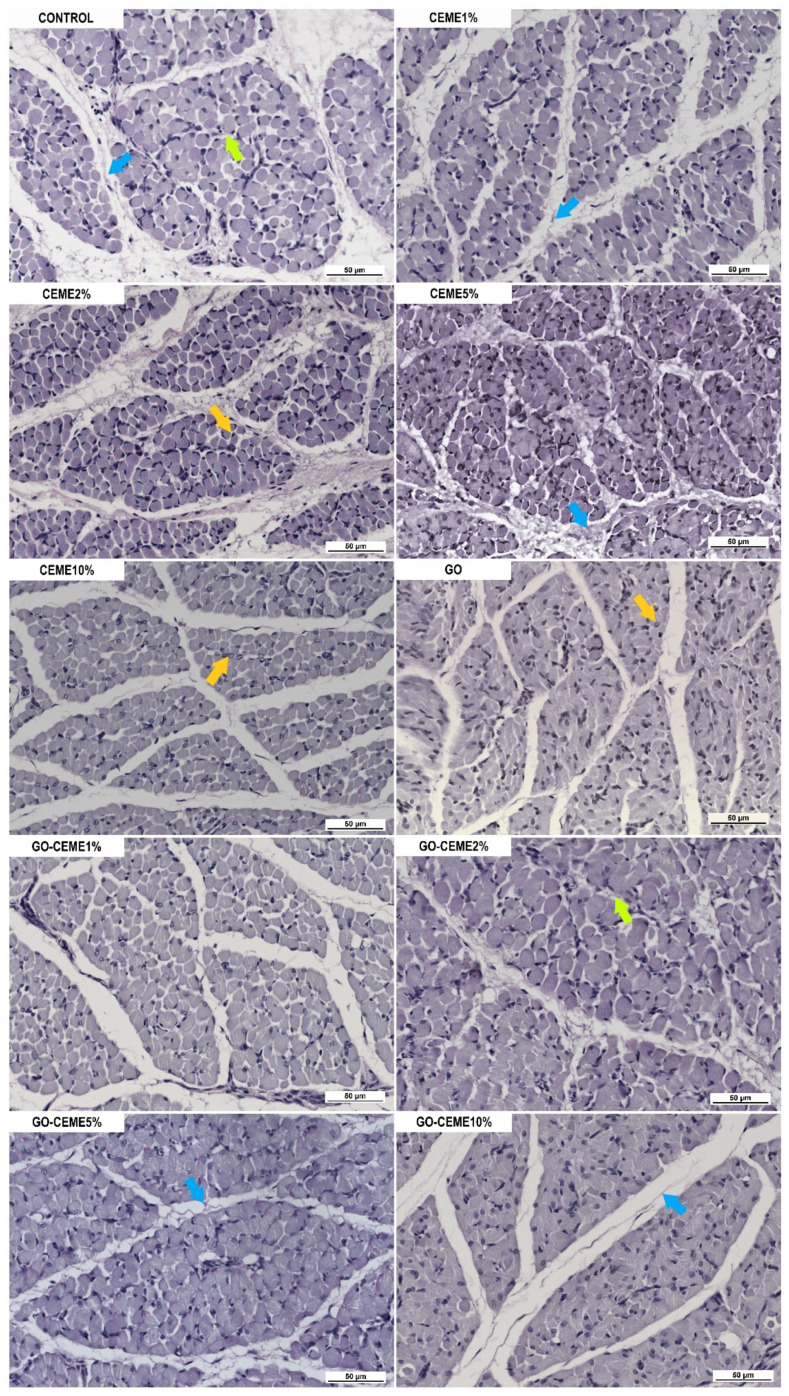
Images of muscle tissue sections after Periodic Acid–Schiff (PAS) staining; yellow arrow–nuclei; green arrow–endomysium; blue arrow–perimysium.

Morphological evaluation of the cross-section of the hindlimb of a 20-day-old chicken embryo showed no pathological changes in the muscle structure in any group. However, in the image of the muscle cross-section, there were significant differences in the muscle structure between the groups (Figure 4). The muscles of the embryos from the control group were characterized by an extensive perimysium with a large amount of connective tissue as well as a clearly marked endomysium, and the cells were loosely packed. In the groups treated with CEME, especially CEME2% and CEME5%, the structure of the muscles were better developed; more densely packed cells, more cell nuclei, and less connective tissue were observed. In the GO and GO-CEME10% groups, the cells were less developed with less marked endomysium, while in the GO-CEME1%, GO-CEME2%, and GO-CEME5% groups to the greatest extent, round, well-developed cells were observed. Summing up, the most favorable structure was characterized by the muscle cells of the embryos from the CEME2% and CEME5% groups, which contained densely packed round cells with a large number of nuclei.

**Figure 5:**
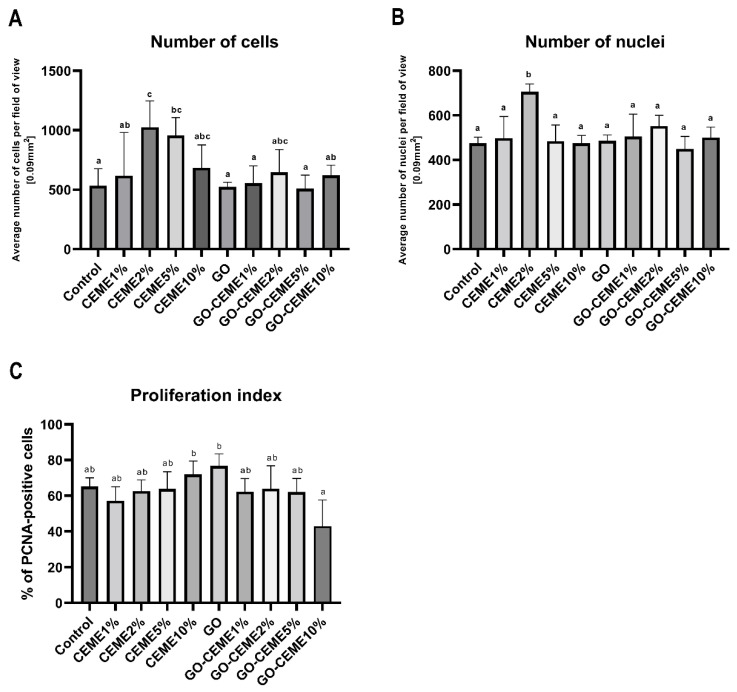
(**A**) Average number of cells counted on microscopic field of view [area 0.09 mm^2^] of specimens after Periodic Acid–Schiff (PAS) staining; (**B**) Average nuclei number counted on the microscopic field of view [area 0.09 mm^2^] of specimens stained with Hematoxylin; (**C**) Proliferation Index presented as percentage of PCNA-positive nuclei to the total nuclei number counted on microscopic field of view [area 0.09 mm^2^] of specimens after immunohistochemical detection of PCNA with Hematoxylin co-staining. Presented as mean value with standard deviation, different letters denote significant difference, *p* < 0.05.

The analysis of immunohistochemical parameters showed a significant increase in the number of cells in the CEME2% and CEME5% groups, calculated on microscopic images per unit area (field of view, 0.09 mm^2^), compared to the other groups (Figure 5A). In the CEME2% group, a greater number of cell nuclei was also observed (Figure 5B). The cell proliferation index, measured by determining the number of PCNA-positive nuclei versus the total number of nuclei, did not increase in any group, compared to the control group. However, compared to GO-CEME10% the proliferation index increased in CEME10% and GO groups (Figure 5C).

### 3.5. SOD Activity

**Figure 6:**
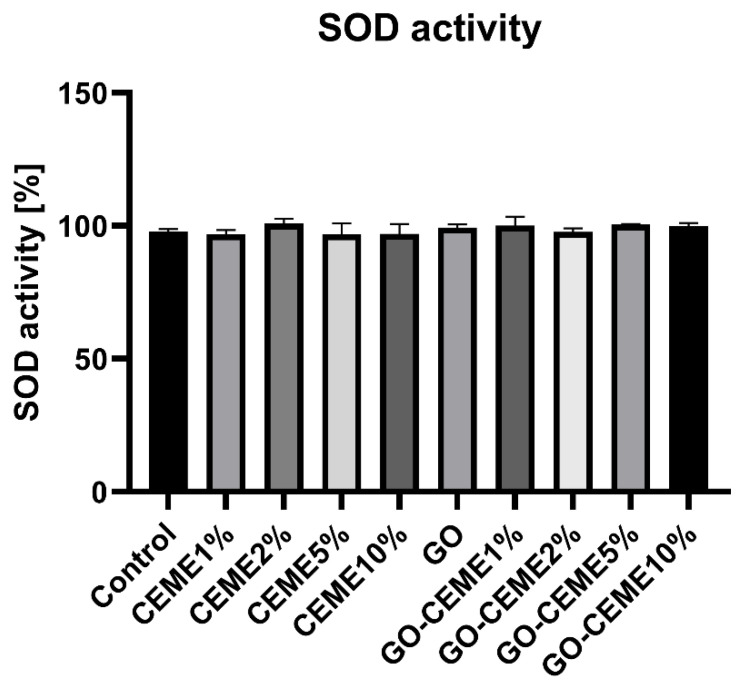
Superoxide dismutase activity in liver samples collected from chicken embryos on 20th day of embryogenesis.

The experiment also measured the activity of superoxide dismutase (SOD) in the liver, a key antioxidant enzyme. No influence of the applied factors on the activity of SOD was observed. The average activity was near 100% in all groups (Figure 6).

## 4. Discussion

In previous studies [9], we used CEME extract to stimulate chicken embryo muscle progenitor cells to create pseudo-muscle tissue in vitro. We obtained a spectacular result because the addition of CEME to the culture medium stimulated spontaneous muscle contraction. This result inspired further research on the use of CEME to improve the structure of the skeletal muscle of a chicken embryo in ovo.

In in vitro experiments, cells derived from the pectoral muscle of a 9-day-old chicken embryo were used. The cells constituted a population of muscle precursor cells as documented by [9]. In previous tests, we assessed the toxicity of CEME using the Trypan blue test, which showed a reduction in the number of cells with CEME by 5% [9]. In the current studies, we obtained a more precise response to the potential toxicity of CEME by testing the effect of the extract on the proliferation of muscle progenitor cells with the BrDU test and their metabolism with the MTT test. The results of the MTT test clearly showed the toxicity of CEME, administered at the level of 10%. The proliferation test, while not showing a negative effect of the administration of CEME10%, was also not found to have a positive effect of CEME10%. In the other studies, an extract from the muscles of a chicken embryo has been used to cultivate nerve cells [18,19,20]. However, a whole chicken embryo (CEE) extract, obtained from an 11-day-old embryo, is usually used to cultivate a variety of cells that require a higher concentration of growth factors than the standard medium [21]. It was found that the use of up to 50 ppm of CEE extract has a positive effect on the culture of fibroblasts derived from chicken embryos [22]. CEE extract is commercially available.

This preliminary in vitro toxicity assessment allowed the choice of CEME concentration for in ovo studies in chicken embryos. CEME concentrations of 1%, 2%, 5%, and 10% for injection into the embryo were selected for further research, thus eliminating only the lowest concentrations, possibly below the effectiveness level.

In previous studies [9], GO was used as a scaffold layer for differentiating muscle cells, while in these studies, graphene flakes were to be a form of CEME transport and protection. The concentration of GO at the level of 100 ppm used in the research was selected based on our own experience and confirmed the biocompatibility of low concentrations of GO [11,13,23,24]. However, in the presented research, a decrease in the metabolic activity of muscle cells was observed when administered to the GO medium, which can be explained by a certain activity of oxygen groups available on the surface of graphene flakes -OH, =O, -COOH [25] and the generation of a small number of reactive oxygen species. The reversible, non-covalent bonds can be created with other molecules, especially with proteins, forming the so-called protein crown. Caused by this, GO can be a carrier of proteins and release these compounds into the environment over time. When analyzing the results of GO-CEME complex administration, we found that the toxicity of GO disappears when combined with CEME proteins, which results from the protein crown effect [26,27]. Moreover, the GO-CEME complex significantly activates the proliferation of chicken embryo muscle cells, which may be related to the gradual dosing of proteins released from the GO surface. The finding of a beneficial effect of GO-CEME complexes administered at the level of up to 5% CEME on cell proliferation confirmed the advantage of GO and CEME complexation [28,29] as a method to improve GO biocompatibility. Most importantly, the complex of CEME and GO significantly increased the positive effect of CEME on cell proliferation. Cell proliferation (BrdU test) was enhanced by the GO-CEME complex compared to the free CEME used and confirmed the choice of GO-CEME1%, GO-CEME2%, and GO-CEME5% for chicken embryo studies. However, considering cell culture is a more sensitive experimental medium than a chicken embryo, so the controversial concentration of GO-CEME10% was also selected.

The basic test for biocompatibility testing of nanomaterials is hemotoxicity. Interestingly, blood cells’ unicellular structures, were not destroyed under the influence of GO and the controversial concentration of CEME10% and the GO-CEME10% complex compared to muscle progenitor cells (MTT test). It seems that cells that adhere to the substrate, such as muscle cells, are much less mobile than rapidly migrating blood cells in the fluid, and therefore the former may be more sensitive to potentially harmful factors. The level of GO hemotoxicity is defined as above 75 μg/mL; however, it can be easily modified by functionalization of GO surface [30] as confirmed by our results,

The potential toxicity of GO, CEME, and their complex was also investigated in in ovo studies on chicken embryos. The chicken embryo is a unique biological model that allows for a quick and precise response; hence, it is used to test drugs [31,32,33] as well as graphene materials [34,35,36]. Analysis of embryo growth and development at the systemic level showed no differences between GO- and CEME-treated embryos and GO-CEME complexes. The analysis of blood biochemical parameters also did not indicate the toxicity of the factors used on the basis of the activity of liver enzymes and albumin concentration. However, it seems that the introduction of an additional amount of protein (CEME) slightly increased the catabolism of the protein, and the unused part of it was deaminated, which is indicated by the increased concentration of urea in the CEME group. Administered L-Arginine supplement in ovo increased the muscle mass of broiler chickens and did not cause an increase in total protein and albumin levels in chickens during the hatching period [37]. This result indicates that the genetic potential of anabolism is inhibited by the limited reserves stored in the egg. However, the excess of supplemented protein is not metabolically beneficial [38]. Therefore, CEME used on the GO platform reduced the tendency to increase the urea concentration in embryos’ blood.

Glucose conversion was also slightly disturbed but not significantly different between groups. The transformation of carbohydrates in the embryo can be modified by the supply of a significant amount of protein (CEME). Glucogenic amino acids could be converted into glucose and glycogen which could be stored in the yolk sac and to a lesser extent in the liver. At the end of embryogenesis, yolk sac glycogen is extensively broken down to glucose 6-phosphate and then converted by G6PC2-to free glucose [39].

In the case of an excessive supply of glucose via glucogenic amino acids, the reserves accumulated in the liver and muscles, as a result of in ovo administration of CEME10% on day 1 of embryonic development, could reduce the glucose release from the yolk sac. Interestingly, a decrease in glucose levels was also observed under the influence of GO. Graphene oxide may react with glucose due to its reducing abilities [40], which in turn may reduce the level of free glucose in the blood. Most of the GO-CEME complexes did not affect the glucose level, which can be explained by the involvement of available oxygen groups by binding to CEME. In addition, CEME derived proteins could also be associated with GO and to a lesser extent be a source of glucogenic amino acids and, consequently, glucose. GO has numerous -OH, =O, -COOH groups on its surface [25]; however, they do not seem to be involved in the oxidation processes, leading to redox imbalance, as evidenced by the lack of changes in SOD superoxide dismutase activity. Studies by other authors found that as GO levels increased, the concentration of H_2_O_2_ decreased, which indicates that GO acts as an electron absorber in the system [41]. Moreover, GO also reacted preferentially with O_2_^−^ [42]. Our studies also did not reveal the pro-oxidative activity of GO. It did not affect the activity of SOD, catalyzing the superoxide radical anion dismutation reaction.

Analyzing the influence of GO, CEME, and GO-CEME complexes on the growth and development of the embryo, we found no increase in the embryo weight. However, the maturation of muscles in the embryonic period is largely due to their hyperplasia, the process of hypertrophy intensifies only after hatching [43]; therefore, the increase in muscle mass on the 18th day of embryogenesis does not have to be noticed. However, the microstructure of the muscle, and especially the number of muscle fibers, should indicate its later condition, which will be fully mature after the hyperplasia stage. The number of muscle cells is fully established by the point of hatching, and embryonic myogenesis is the crucial process that determines the muscle mass during the slaughter of chickens [39], so the assessment of the muscle structure on day 20 is fully justified.

Histological analysis of the cross-section of the muscles of the chicken embryo on day 20 showed that in ovo administration of GO did not positively affect the maturation of the hindlimb muscles of the embryo, which was probably associated with the formation of a protein crown on the surface of the GO flake, which could permanently bind certain proteins and be the cause of their deficiency. Furthermore, GO-CEME10% caused the deterioration of the muscle structure, which may be related to the excessively high level of CEME administered (10%). What is more, the excess peptides related to GO could not be degraded. Therefore, the administration of CEME10% was safer.

The most favorable morphological image of the embryonic muscles (well-developed numerous cells and less connective tissue), supported by morphometry analysis (number of cells), was observed after administration of the CEME extract at the level of 2 and 5%. However, only the addition of CEME2% increased the number of cell nuclei.

It could be noted that supplementation of CEME at day one of injection could be effective later on. However, proteins are also a source of smaller peptides and amino acids, constituting a reserve pool of potentially deficient components for protein building during the following days of embryogenesis. The process of proliferation and differentiation of muscle cells from the pool of premyoblastic targets starts at the beginning of embryogenesis. Expression of Myf5 is already observed at HH stage 9, so CEME injected into the albumen at HH stage 3 could be used by these cells from the very beginning. Myotomal cells, embryonic myoblasts, and fetal myoblasts, having at their disposal a unique protein cocktail, could fully exploit their genetic potential, normally limited to spare material accumulated in the egg. CEME extract was a source of key enzyme proteins involved in energy production, characteristic of the period of the final stage of development of a chicken embryo. The top 100 identified proteins included proteins involved in glycolysis such as Glucose-6-phosphate isomerase (GPI), Phosphoglycerate kinase 2 (GPI), Glyceraldehyde-3-phosphate dehydrogenase (GAPDH), Phosphoglycerate kinase 2 (PGK2), Fructose-bisphosphate aldolase (ALDOC), alpha-enolase (ENO1), Phosphoglycerate mutase 1 (PGAM1), and Triosephosphate isomerase (TPI1). The presence of these enzymes could also reduce the level of glucose in the embryo’s blood (CEME10% group) as a result of the intensification of glycolysis. Glucose is present in the yolk in a very small amount (about 1%) and is used as an energy source in the initial phase of embryogenesis and during the hatching [39,44], another source of energy are proteins, and only then are fats used [39]. Thus, the beginning of embryogenesis is an energetically difficult stage, taking into account the low level of glucose and the energetically unfavorable process of obtaining energy from protein. CEME contained the enzymes of ATP synthesis in the mitochondrion ATP synthase subunits A, B, and D (ATP5F1A, ATP5F1B, ATP5F1D) and proteins involved in the synthesis of high-energy compounds such as Isocitrate dehydrogenase NADP (IDH1 I IDH2), Nucleoside dip (IDH2), and Nucleoside synthesis of nucleoside triphosphates other than ATP. Supplementation of such a powerful dose of the enzyme apparatus allowing the production of energy in the muscles, and especially the proteins involved in oxidative phosphorylation, could stimulate muscle development. Moreover, the pool of proteins provided by CEME was also a source of glucogenic amino acids, which could spare the own protein accumulated in the egg. The increase in blood urea level would confirm the deamination of amino acids. The energy that, thanks to supplementation, could be produced in the early stage of embryonic development could be used for the synthesis of the material necessary for the intensive proliferation of myogenic cells in the earlier stages of embryonic development.

Moreover, CEME was the source of proteins involved in the formation of muscle structure, including proteins related to the extracellular matrix. First of all, it contained the key, multifunctional Protein disulfide isomerase (P4HB), among others responsible for the collagen fibril organization [45]. Another one was SerpinH1 (SERPINH1), specifically binding to collagen [46]. CEME also contained the Collagen alpha-1 (XIV) chain structural protein responsible for the collagen organization, including the cell-cell adhesion and ECM organization. It also contained structural proteins such as Desmin (DES) and Vimentins (VIM)-type III intermediate filaments, the first specific for muscles and shaping their normal structure [47,48]. In addition, the presence of other proteins involved in cytoskeleton formation was observed, such as Spectrin alpha chain (SPTAN1 and SPTBN1) and the most conserved Actin (ACTG1) forming a contractile apparatus.

Proteomic analysis showed a significant contribution to the CEME of nervous system proteins. For specific neuronal development and polarity, Dihydropyrimidinase-related protein 2 (DPYSL 2) is responsible for axon elongation and also interacts with microtubules, actin, and tubulin [49,50]. Muscle development is strongly stimulated by the nervous system, so it can be assumed that the presence of this protein could also indirectly stimulate muscle maturation.

## 5. Conclusions

The research concerned the possibility of improving the muscle structure by using CEME extract from physiologically mature muscles and GO flakes as a carrier of the extract, administered in ovo. Basic in vitro studies have documented the biocompatibility of CEME and the GO-CEME complex administered at concentrations 1–5%. No toxicity was observed in in ovo studies. Analysis of the structure of the striated muscle of the 20-old embryo showed a significant improvement, especially the increased number of cells and cell nuclei after administration of 2% CEME. However, GO, although not toxic, did not increase the effectiveness of the CEME extract activity. The research clearly shows the advisability of using the CEME extract as a stimulator of muscle development in the embryonic period. Potential effects on muscle development in broiler chickens require further research.

## Figures and Tables

**Table 1:** Mean body and organ weight of 20-day-old chicken embryos, presented as mean value ± standard deviation.

Group	Egg Mass [G]	Embryo Body Weight [G]	Heart Weight [G]	Liver Weight [G]
Control	54.1 ± 4.42	45.1 ± 3.52	0.23 ± 0.014	0.63 ± 0.063
CEME1%	53.7 ± 2.95	44.5 ± 2.46	0.23 ± 0.043	0.67 ± 0.094
CEME2%	52.6 ± 3.05	43.9 ± 2.32	0.24 ± 0.071	0.61 ± 0.103
CEME5%	52.9 ± 2.70	44.2 ± 2.72	0.23 ± 0.032	0.62 ± 0.092
CEME10%	55.6 ± 3.25	45.6 ± 3.28	0.24 ± 0.024	0.59 ± 0.091
GO	54.8 ± 3.55	47.0 ± 5.49	0.22 ± 0.023	0.63 ± 0.142
GO-CEME1%	51.9 ± 3.49	43.0 ± 2.98	0.23 ± 0.028	0.64 ± 0.085
GO-CEME2%	55.8 ± 3.35	51.2 ± 2.71	0.19 ± 0.072	0.57 ± 0.067
GO-CEME5%	51.2 ± 3.41	47.5 ± 3.57	0.22 ± 0.027	0.65 ± 0.100
GO-CEME10%	52.9 ± 2.74	44.4 ± 1.78	0.24 ± 0.035	0.63 ± 0.081

**Table 2:** Biochemical parameters of blood serum from 20-day-old chicken embryos, presented as mean value ± standard deviation, different superscript a, b denotes significant difference, *p* < 0.05.

Group	Albumins [G/L]	ALT [U/L]	AP [U/L]	AST [U/L]	Total Protein [G/L]	Creatinine [µmol/L]	Urea [Mmol/L]	Globulins [G/L]	LDH [U/L]	Triglyceride [Mmol/L]	Glucose [Mmol/L]
Control	3.15 ± 0.663	3.38 ± 1.230	2738 ±320.2	214.2 ± 52.80	13.3 ± 1.72	19.6 ± 12.19	3.56 ± 0.861 ^ab^	10.1 ± 1.07	1100 ± 84.2	0.71 ± 0.203	14.2 ± 0.30
CEME1%	4.05 ± 1.011	2.68 ± 1.681	2858 ± 1267.2	180.7 ± 62.41	15.8 ± 2.18	28.5 ± 4.47	4.06 ± 0.333 ^ab^	11.7 ± 1.18	1156 ± 343.1	1.08 ± 0.372	12.4 ± 0.64
CEME2%	3.01 ± 0.285	2.52 ± 0.143	2218 ± 176.5	171.5 ± 21.07	12.1 ± 0.85	29.9 ± 1.98	3.94 ± 0.534 ^ab^	9.1 ± 0.57	909 ± 76.3	0.84 ± 1.345	12.3 ± 0.28
CEME5%	3.87 ± 0.413	3.92 ± 1.502	2844 ± 521.9	214.9 ± 56.96	15.5 ± 1.10	28.1 ± 6.18	4.13 ± 0.645 ^b^	11.6 ± 0.82	1213 ± 279.9	1.01 ± 0.231	12.4 ± 0.53
CEME10%	3.42 ± 0.142	3.58 ± 2.343	2249 ± 309.6	202.6 ± 118.14	13.3 ± 1.02	37.3 ± 18.31	4.17 ± 0.912 ^b^	10.0 ± 0.95	1029. ± 542.3	1.22 ± 1.212	12. ± 1.65
GO	3.52 ± 0.522	4.52 ± 0.786	2199 ± 439.4	299.4 ± 29.25	13.6 ± 1.28	30.6 ± 6.12	4.19 ± 0.583 ^b^	10.1 ± 0.88	1448 ± 389.0	1.02 ± 0.343	11.55 ± 1.20
GO-CEME1%	3.15 ± 0.217	3.05 ± 1.345	2161 ± 377.5	200.3 ± 3.25	12.9 ± 0.85	23.6 ± 2.33	3.31 ± 0.034 ^ab^	9.8 ± 0.64	1189 ± 269.5	1.36 ± 0.465	14.7 ± 1.27
GO-CEME2%	3.68 ± 1.131	3.68 ± 1.50	2629 ± 939.1	200.6 ± 51.81	15.0 ± 3.67	26.1 ± 4.29	3.75 ± 0.593 ^ab^	11.3 ± 2.60	1117 ± 187.7	0.64 ± 0.161	12.0 ± 0.68
GO-CEME5%	3.9 ± 0.911	2.5 ± 1.55	2496 ± 403.6	190.0 ± 46.74	15.0 ± 2.13	22.8 ± 3.16	3.04 ± 0.200 ^a^	11.1 ± 1.27	1177. ± 289.1	1.04 ± 0.384	14.5 ± 0.74
GO-CEME10%	3.13 ± 0.394	3.71 ± 1.61	2732 ± 707.2	170.4 ± 3.92	12.7 ± 1.49	22.8 ± 2.61	3.37 ± 0.445 ^ab^	9.6 ± 1.21	1130 ± 365.6	1.04 ± 0.472	11.9 ± 1.57

## Data Availability

The datasets used and/or analysed during the current study are available from the corresponding author on reasonable request.
